# Supplementary material for: The Evaluation of the Four-Chamber Cardiac Dissection Method of the Fetal Heart as an Alternative to Conventional Inflow–Outflow Dissection in Small Gestational-Age Fetuses
Source: Diagnostics (Basel). 2022 Jan 17;12(1):223. doi: 10.3390/diagnostics12010223 (PMC8775121; doi:10.3390/diagnostics12010223)
Supplement: Supplementary file 1 [file diagnostics-12-00223-s001.zip › diagnostics-1455207-supplementary.pdf]

Supplementary Materials

Supplementary Table S1. Description of acquisition parameters adapted to the characteristics and size of each case - on the sagittal section**.**

Number Case	Effective Echo Time (TE) (ms)	Repetition Time (TR) (ms)	Slice Thickness (mm)	Field of View (mm)	Matrix (Data Points)	Resolution (cm/pixel)	Number of Slices Acquired	Scanning Time (min)
1	36	7714.4	0.5/075	4.9	384/384	0.0154/0.0149	59	30
2	36	4747.3	0.5/075	4.1	384/384	0.0107/0.0104	38	18
3	36	5934.1	05/075	4.73	384/384	0.0123/0.0121	48	23
4	36	6527.6	05/075	6	384/384	0.0156/0.0131	52	26
5	36	5696.8	0.6/0.85	4.75	384/384	0.0124/0.0125	48	22.47
6	36	9494.6	05/075	5.95	384/384	0.0155/0.0151	70	37.58
7	36	5340.7	05/075	4	384/384	0.0104/0.0060	40	21.21
8	36	6646.2	0.5/0.75	3.93	384/384	0.0102/0.0123	55	26.35
9	36	5934.1	05/075	3.73	384/384	0.0097/0.0104	44	23
10	36	5934.1	05/075	3.73	384/384	0.0097/0.0104	44	23
11	36	6646.2	05/075	2.73	384/384	0.071/0.0112	56	26.35
12	36	5104.4	0.6/0.85	4	384/384	0.0104/0.0104	38	20.24
